# Wavelet transform-based multipitch estimation in polyphonic music

**DOI:** 10.1016/j.heliyon.2020.e03243

**Published:** 2020-01-29

**Authors:** Neeraj Kumar, Raubin Kumar

**Affiliations:** a Department of Electronics Engineering, IIT(ISM), Dhanbad, Jharkhand, 826004, India; b Talentica Software (I) Pvt. Limited, Pune, India

**Keywords:** Electrical engineering, Modified morlet wavelet (MMW), Multipich estimation (MPE), Autocorrelation, Continuous wavelet transform (CWT), Short-time Fourier transform (STFT)

## Abstract

In this paper, a Wavelet transform-based approach for estimation of multipitch in music signal has been proposed. Among the Morlet Wavelet (MW), Mexican hat, and Shannon wavelet that belong to the widely used wavelets in different applications, the Morlet wavelet performs well for estimation of pitch in polyphonic music signals. This is why a method involving modification of the Morlet wavelet has been proposed for achieving better accuracy in estimation of multiple pitches in polyphonic music. Performance of the Modified Morlet Wavelet (MMW) based Multipitch Estimation (MPE) scheme has been compared with that of a method based on Fast Fourier Transform and another based on the original Morlet Wavelet, in terms of percentage Gross Pitch Error (GPE). Piano chord data base and Standard music IOWA data base have been used for performance evaluation of the proposed scheme. Simulation results show that percentage error in pitch (described by the fundamental frequency) is minimum for the proposed i.e. MMW-based method.

## 1 Introduction

Pitch estimation is one of the most widely investigated areas of Music Signal Processing. It is used for music transcription, music data acquisition, chord estimation etc. Music signals can be categorized as monophonic and polyphonic. Monophonic music is simplest in textures and can be easily realized using single note at a time i.e. the signal has single frequency at a time whereas, a polyphonic music signal has multiple frequencies at a time. Pitch finding in monophonic music signals is simple though harmonics of fundamental frequencies are also present with low amplitude in the time domain. In contrast to this, pitch detection in polyphonic music is difficult due to presence of combination of frequencies, corresponding to various notes, in the spectrum thus making it difficult to analyze, especially in the case of identifying two pitches related by an interval of one octave (e.g. a middle C and the next highest C played together).

Several approaches have been reported in the literature for multipitch estimation. Yeh et.al [1] presented a frame based system for estimating the multiple fundamental frequencies of music signal based on short time fourier transform (STFT). A method for estimating the fundamental frequencies of several concurrent sounds in polyphonic music and multiple-speaker speech signals is presented in [2]. The estimation of multiple concurrent pitches in piano recordings is presented in [3, 4, 5, 6] and the piano multipitch estimation using sparse coding embedded deep learning is presented in [7]. Polyphonic transcription method converts a music audio signal into a human-readable musical score by integrating multi-pitch detection and rhythm quantization methods [8]. The direct magnitude spectrum analysis algorithm in [9] can also detect F_0,_ and is applicable to polyphonic signals. Many of the available methods for music transcription are based on extraction of the fundamental frequencies using frequency analysis tools such as Fast Fourier transform (FFT) [10, 11, 12]. In [13], a method for automatic transcription of music signals based on joint multiple-F_0_ estimation is presented. In [14], a multi-pitch analysis method using specmurt analysis with iterative estimation of the quasi-optimal common harmonic structure function is described. A systematic evaluation of MPE that emphasizes generalizability and robustness is presented in [15]. In [16], a multiple pitch estimation algorithm using non-homogeneous poisson processes for the automated multiple-pitch estimation problems is proposed.

Pitch estimation methods can be classified into four categories [17], depending on the domain of analysis: (1) temporal (2) frequency (3) temporal-frequency, and (4) statistical. The autocorrelation function that is widely used in the methods falling under the first category is a temporal pitch estimator that measures similarity between the signal and translated (shifted) version of itself. A number of methods belonging to the second category-i.e. involving analysis in the frequency domain have been already mentioned in the preceding paragraph. Considering the advantages of methods using time-frequency analysis (especially when wavelets are used for such analysis) over the methods belonging to the first two categories, an approach that falls under the third category has been proposed here. The wavelet transform that is essentially convolution of a signal f(t) with a dilated and translated version of a function ѱ(t) (the mother wavelet that needs to fulfil certain prerequisites) [18] can provide good time as well as frequency localization which is a distinct advantage over others and hence it is extremely useful for extraction of features in music signals where signal properties vary over time. Some of the works on Wavelet transform that are relevant to the present work are mentioned in the following paragraph.

Mallat and Zhong [19] proved that the dyadic wavelet transform exhibits local maxima at the point of sharp variation of the signal. Chan et al [20] and Jehan [21] presented the estimation of local maxima using wavelets to find the pitch period. In [22], an approach called empirical wavelet transform (EWT) is presented with wavelet filter bank adapted to the processed signal. A Complex continuous wavelet transform (CCWT) estimates the fundamental frequencies in single-channel polyphonic signals were proposed in [23]. A modified pitch detection method based on complex Morlet wavelet is presented in [24]. In [25] a fundamental frequency determination method dependent on the autocorrelation compression of the multi-scale product of speech signal is presented. In [26] a wavelet based method proposed and compared with auto-correlation methods for pitch period estimation.

This paper introduces a reasonably straightforward and computationally efficient fundamental frequency (F_0_) estimator for polyphonic music signals. After making study related to performances of a set of wavelets in pitch estimation in music signals, the Morlet wavelet is found to perform the best in terms of accuracy of pitch. Hence, an attempt was made to achieve still higher accuracy with a novel wavelet transform – based pitch detection technique that involves some modification of the original Morlet wavelet.

The rest of the paper is organized as follows: pitch estimation in polyphonic music is discussed in Section 2. Section 3 presents the simulation results along with comments on them. Finally section 4 concludes the paper.

## 2 Pitch estimation in polyphonic music

### 2.1 Methodology for pitch estimation

**Figure 1:**
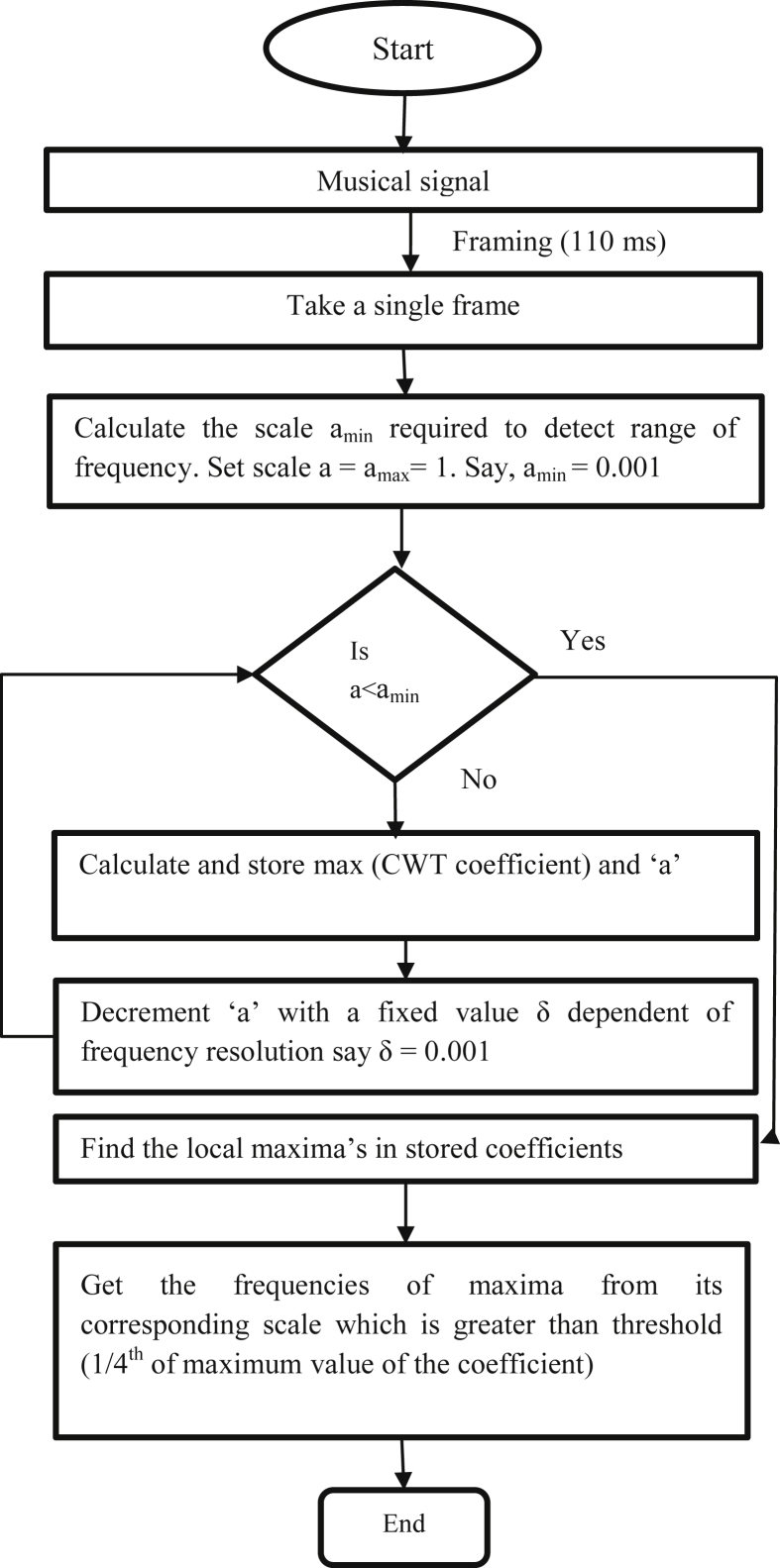
Flow chart of Multipitch detection.

Polyphonic music is complex as it is having multiple notes at a time and this signifies the presence of multiple pitch at a time. The multiple pitch detection algorithm flow chart is shown in Figure 1. The heart of the algorithm is the selection of wavelet that satisfies the two conditions:

(1) The wavelets should have bandpass nature
(2) Bandwidth of wavelets should be small at lower scale

The algorithm finds the dominant frequency contents present in the musical signal with the help of suitable wavelet. The selected wavelet detects the close frequency that separates the notes in musical signal.

### 2.2 Mathematical justification of pitch detection algorithm with morlet wavelet

All the wavelets represent specific frequency at a specific scale. But for applications like pitch detection, it is required to have wavelets with smaller bandwidth at lower scales. Since at lower scale the time domain signal contracts and that leads to frequency domain expansion, the Morlet wavelet can be used for the detection of multiple pitch.

Consider a signal f (t) consisting of multiple frequencies f_1_, f_2_……..

(1) f (t) = sin(2πf_1_t) + sin(2πf_2_t) + ..........

The wavelet function Ψ(t) for Morlet can be expressed as [18]. $\text{Ψ}\left(\text{t}\right)=e^{-{\left(\alpha t\right)}^{2}}\text{cos}\left(\sqrt{\pi \frac{2}{ln2}}t\right)$.

(2) $$\text{Ψ}\left(\text{t}\right)={\text{e}}^{-{\left(\text{αt}\right)}^{2}}\;\cos\left(2{\text{πf}}_{\text{i}}\text{t}\right)$$

which can be written as: where ‘α’ is the inverse of scale and ${\text{f}}_{\text{i}}=\sqrt{\frac{1}{2\text{ln}2}}$ is a constant. The Fourier transform of the wavelet function (exponential part of the wavelet),

(3) $$\text{F }\left[{\text{e}}^{-{\left(\text{αt}\right)}^{2}}\right]=\frac{1}{\text{α}\sqrt{2}}{\text{e}}^{-{\frac{\text{f}}{4{\text{α}}^{2}}}^{2}}$$

(4) $$F\left[\cos\left(2{\text{πf}}_{\text{i}}\text{t}\right)\right]=\;\sqrt{\frac{\text{π}}{2}}\left[\delta \left(f-2\pi f_{i}\right)+\delta \left(f+2\pi f_{i}\right)\right]$$

Therefore, Fourier transform of the wavelet function,

(5) $$\text{F}\left[\text{ψ}\left(\text{t}\right)\right]=\frac{1}{2\text{α}}\left[{\text{e}}^{-{\frac{\left(\text{f}+2{\text{πf}}_{\text{i}}\right)}{4\alpha^{2}}}^{2}}+{\text{e}}^{-{\frac{\left(\text{f}-2{\text{πf}}_{\text{i}}\right)}{4\alpha^{2}}}^{2}}\right]$$

Thus in frequency domain the Morlet wavelet will appear as a Gaussian at different frequency points for different scales. Similarly if we consider the signal to be a combination of different sinusoids then it will appear as an impulse a different frequency location, depending on the frequency contents present in the signal.

Fourier transform of (1) will be

(6) $$F\;\left[f\left(t\right)\right]\;=\;j\sqrt{\frac{\pi }{2}}\left[\delta \left(f-2\pi f_{1}\right)+\;\delta \left(f-2\pi f_{2}\right)+\ldots \ldots \ldots \ldots ..-\;\delta \left(f+2\pi f_{1}\right)-\;\delta \left(f+2\pi f_{2}\right)-\ldots \ldots \ldots \right]$$

The musical signal is convolved with the wavelet. If the frequency present in the musical signal corresponds to the scale of wavelet (centre frequency) then a Gaussian will appear. The CWT coefficient values will be maximum at these points. Finally by detecting these maxima points and its corresponding scales we can easily detect the pitch present in the music signal. In the Fourier transform of Morlet wavelet if the factor ‘α’ will be more then bandwidth will be more. So modification in the wavelet should be such that the bandwidth remains less. It can be achieved either by scaling (using exponential) or modifying wavelet such that the exponential power is not varied as square of scale. This modification is done in the next section to detect the multiple pitches.

### 2.3 Wavelet for pitch detection

The modified morlet wavelet can be used to detect multiple musical pitch. Modification is done only in the exponential part which leads the change in wavelet at every scale. The modification was done such that the wavelet should appear with time as $e^{\frac{-t^{2}}{a}}$ with scale ‘a’. The reason behind this was the frequency domain behavior of the wavelet with scale. The actual Morlet wavelet is having larger bandwidth in the frequency due to inverse of square of scale present in its Fourier transform. Due to this it is unable to detect the closely separated frequency signals. Keeping this in mind the new wavelet uses $e^{-\left|t\right|}$ to restrict the wavelet in time domain. The scale ‘a’ is associated with time ‘t’. The proposed wavelet is given below.

(7) $$\Psi \left(t\right)\;=\;e^{-\left|t\right|}cos\left(2\pi t\right)$$

#### 2.3.1 Frequency response of the wavelet

The Fourier domain representation of the proposed wavelet can be seen as convolution of two parts as shown for other wavelets.

(i) Fourier transform of $e^{-\left|t\right|}$
(ii) Fourier transform of cos(2πt)

The Fourier domain representation can be given as

(8) $$F\left[\Psi \left(t\right)\right]\;=\;\frac{\sqrt{\frac{2}{\pi }}}{4\pi^{2}f^{2}+1}\text{ ∗}\sqrt{\frac{\pi }{2}}\left[\delta \left(f-2\pi \right)+\;\delta \left(+2\pi \right)\right]$$

or,

(9) $$F\left[\Psi \left(t\right)\right]\;=\;\frac{1}{4\pi^{2}f^{2}+1}\text{ ∗ }\left[\delta \left(f-2\pi \right)+\;\delta \left(f+2\pi \right)\right]$$

Considering a constant ‘k’ (k > 1) in the exponential part of the wavelet as

(10) $$\Psi \left(t\right)=e^{-\left|\frac{t}{k}\right|}cos\left(2\pi t\right)$$

Now the Fourier transform of the wavelet Ψ(t) can be given as

(11) $$\text{F}\left[\text{Ψ}\left(\text{t}\right)\right]\;=\;\frac{\text{k}}{{\left(2\text{πfk}\right)}^{2}+1}\text{ ∗ }\left[\text{δ}\left(\text{f}-2\text{π}\right)+\text{ δ}\left(\text{f}+2\text{π}\right)\right]$$

Note: - ‘*’ denotes the convolution.

With the factor ‘k’ the wavelet in time domain will expand but it leads to frequency domain contraction. It is necessary when we are considering the detection of multiple pitches because as the scale decreases the bandwidth will increase. So we need to have a factor ‘k’ such that the wavelet will be able to track the individual notes present in the signal. However as we move from lower notes to higher notes the frequency separation between two consecutive notes increases gradually but with presence of square of scale (k^2^) in the transformed domain expression the bandwidth expands at much lower scales and will not track the closely frequency separated signals. So value of'k’should be greater than 1 so that at lower scales it can help to have a smaller bandwidth.

#### 2.3.2 Graphical representations of wavelet and its magnitude response

**Figure 2:**
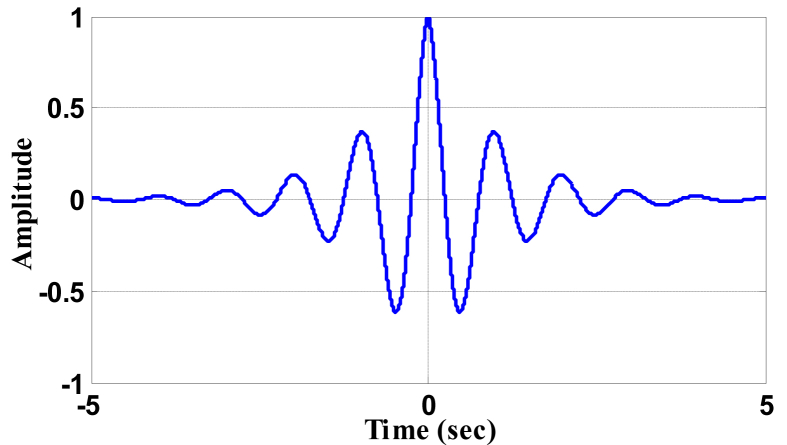
Time domain plot of wavelet ($e^{-\left|t\right|}cos\left(2\pi t\right)$).

**Figure 3:**
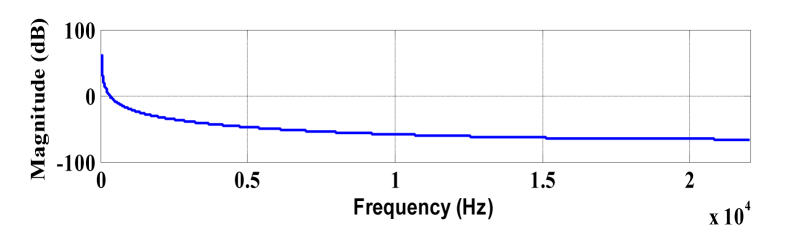
Magnitude response of wavelet at scale a = 0.1 (center frequency = 10 Hz).

The time domain wavelet can be seen in Figure 2 with its magnitude response in Figure 3. The waveform has been plotted by considering sampling frequency 44.1 KHz. This wavelet has (-5 5) as effective support (see Figure 3).

**Figure 4:**
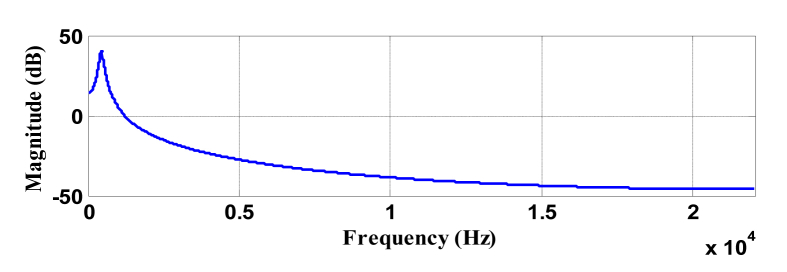
Magnitude response of wavelet at scale a = 0.01 (center frequency = 100 Hz).

**Figure 5:**
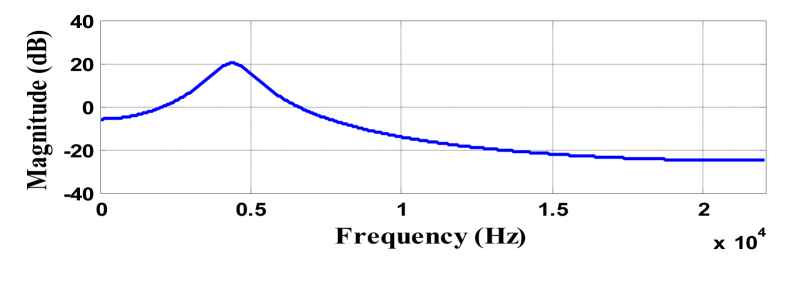
Magnitude response of wavelet at scale a = 0.001 (center frequency = 1000 Hz).

The simulation is performed on MATLAB^(R)^ for both simulated signals and instrumental musical signals [27, 28]. Frequency resolution has been set to 1 Hz i.e., the scale is decremented such that wavelet center frequency changes to ~0.85 Hz at each scale change. The simulation is carried out at a single frame of 110 ms. Plotting function uses scale to frequency conversion formula [29] described in Eq. (12).

(12) $$f_{a}=\frac{f_{c}}{a\times T}$$

where, f_a_ is the pseudo frequency of scale ‘a’, f_c_ is the centre frequency and ‘T’ is the sampling time. Presence of the frequency content in the signal will appear as a peak when the scale of the wavelet represents the same frequency present in the signal. Figures 4 and 5 represent the frequency response of selected wavelet at different scales.

Clearly with the change of scales, change in wavelet's central frequency is observed. It is also observed that at lower scale, the wavelet's frequency response is of lesser bandwidth as discussed earlier (in section 2.3.1).

By using the simulation in matlab, we found that peaks are present at frequencies where pitch is present. So our purpose is the pitch estimation, which is acceptable in Morlet wavelet but other wavelets (Shannon or Mexican Hat) are not suitable for the pitch.

As a matter of fact the Morlet wavelet can be used to detect the multiple pitch. Modification is done in exponential part which leads the change in wavelet at every scale.

## 3 Simulation results

The simulation results are evaluated for four note polyphonic signals. These signals are synthetically generated using the musical instrument at the University of Iowa [27].The fundamental frequency of these synthetically generated musical signals are as follows: (1) F_01_ = 130.81Hz (C3), F_02_ = 146.83Hz (D3), F_03_ = 164.81Hz (E3) and F_04_ = 155.56Hz (Eb3).

**Figure 6:**
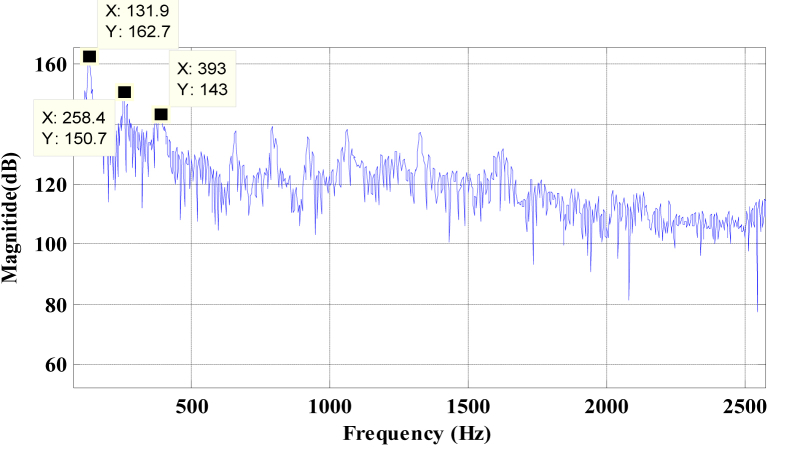
Plot of FFT coefficient Vs frequency for Bass Flute C3 audio.

There are no harmonic relations between these frequencies.Figure 6 shows the frequencies at which peaks occurred that also represent the pitches present in the signal.

**Figure 7:**
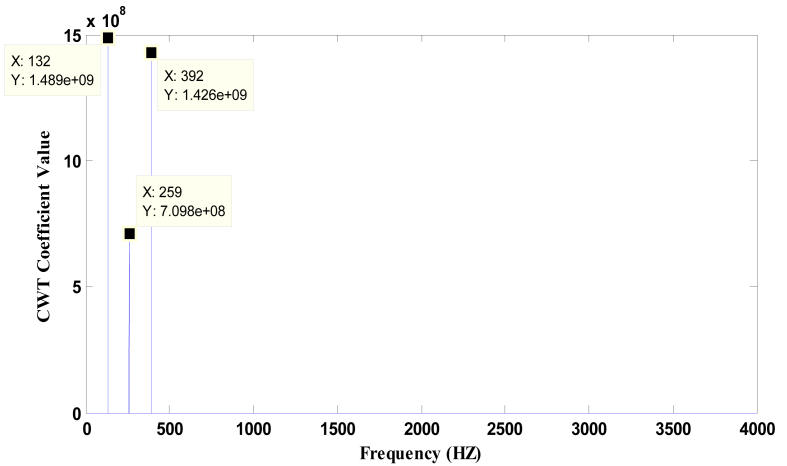
Plot of CWT coefficient Vs frequency for Bass Flute C3 audio using Original Morlet Wavelet.

**Figure 8:**
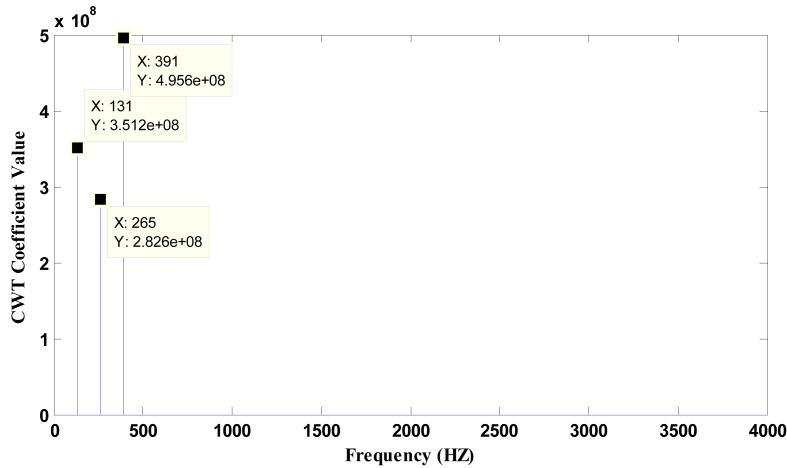
Plot of CWT coefficient vs. frequency for Bass Flute C3 audio using Modified Morlet Wavelet.

Figures 7 and 8 represent the dominant frequencies with their maximum CWT value. The lower coefficients are neglected by selecting a proper threshold value. The chosen threshold value is 1/4^th^ of the CWT coefficient value.

The theoretical frequency of the given note is calculated using $f_{n}=f_{0}\times a^{n}$, where *f*_*0*_ is the frequency of one fixed note, *n* is the number of half steps away from the fixed note, *f*_*n*_ is the frequency of the note *n* half steps away and *a* = (2)^1/12^ [30].

From the Table 1 it is observed that the pitch detection error remains less than 3% that satisfies the acceptable pitch detection error of 3% [31]. Moreover, the proposed method produces better results than the FFT in terms of gross pitch error.

**Table 1:** Frequency Obtained for Bass Flute (Using methods -FFT, Original Morlet Wavelet and Modified Morlet Wavelet) from Data-base.

Notes	Theoretical freq ($f_{n}$ in Hz)	Frequency obtained using FFT	Frequency obtained using Morlet Wavelet (MW)	Frequency obtained using modified Morlet Wavelet (MMW)	Gross pitch error Theoretical vs. FFT)	Gross pitch error (Theoretical vs. MW)	Gross pitch error (Theoretical vs. MMW)
C3	130.81	131.9	132	131	-1%	-0.91%	0%
	261.62	258.4	259	265	1%	1.00%	-1%
	392.43	393	392	391	0%	0.11%	0%
D3	146.83	148	147	146	-1%	-0.12%	1%
	293.66	288	291	290	2%	0.91%	1%
	440.49	436	431	426	1%	2.15%	3%
	555.24	554.5	549	541	0%	1.12%	3%
E3	164.81	164.2	163	163	0%	1.10%	1%
	329.62	317.6	327	324	4%	0.79%	2%
Eb3	155.56	156.1	155	154	0%	0.36%	1%
	311.06	306.8	306	307	1%	1.63%	1%
	466.59	465.7	462	456	0%	0.98%	2%

**Figure 9:**
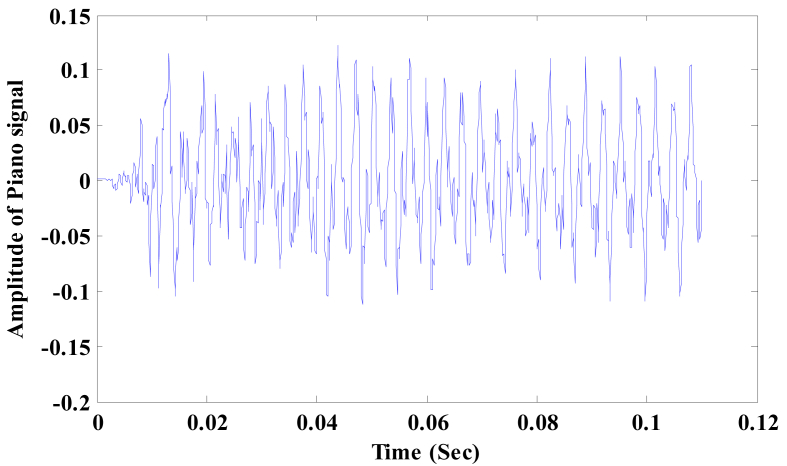
Piano signal playing B-minor chord.

**Figure 10:**
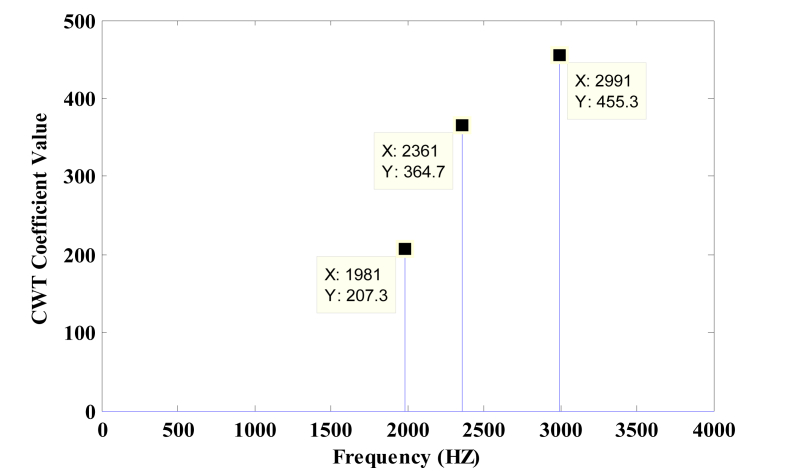
Pitch obtained for piano signal playing B-minor chord.

Figure 9 shows the waveform of ‘B-minor chord’ played by a piano instrument having sampling frequency of 44.1 KHz with a frame size of 110 ms. Figure 10 shows the B-minor chord frequencies (1981Hz, 2361Hz, 2991Hz) obtained by modified morlet wavelet which are in close proximity to the theoretical frequencies (1979.5 Hz, 2349.3 Hz and 2960 Hz). Clearly, the simulated results agree with the theoretical values.

The time complexity for the algorithm depends on CWT at each scale. At each scale the maximum value of coefficient is calculated in parallel with CWT. As reported in [32] the complexity of CWT is O (N). If we consider the scale change along with the CWT, then the complexity for the calculation of maximum CWT coefficients would be O(N) [ O(N) + O(N)] = O(Nˆ2). However the Computational complexity of the FFT for length N is O(N*$log_{2}N$).

For the instrument of piano, the frequency of notes should be similar in the every chord played. All notes involved in the chords related to 6^th^ octave and the corresponding note frequencies are shown in Tables 2 and 3. Every note for different chord is playing the same frequency that verifies that the modified morlet wavelet is able to detect the multiple pitches present in the signal.

**Table 2:** Frequency obtained for minor chords of piano with FFT, morlet wavelet (MW) and modified morlet wavelet (MMW).

Sl. No.	File Name	Chord Played	Notes Present	Theoretical frequencies of notes ($f_{n}$)	Frequencies Detected (Hz) with FFT	Frequencies Detected (Hz) with MW	Frequencies Detected (Hz) with MMW
1.	Minor C	C	C6, D6#, G6	1046.5, 1244.5, 1568	1047,1244,1572	1048, 1244, 1569	1047, 1244, 1570
2.	Minor C#	C#	C6#, E6, G6#	1108.7, 1318.5, 1661.2	1106,1314,1672	1109, 1316, 1672	1109, 1316, 1671
3.	Minor D	D	D6, F6, A6	1174.7, 1396.9, 1760	1168,1392,1755	1177, 1394, 1761	1176, 1393, 1761
4.	Minor D#	D#	D6#, F6#,A6#	1244.5, 1480, 1864.7	1246,1480,1871	1244,1478, 1872	1246,1479, 1870
5.	Minor E	E	E6, G6, B6	1318.5, 1568, 1979.5	1311,1564,1986	1315, 1570, 1983	1316, 1565, 1979
6.	Minor F	F	F6, G6#, C7	1396.9, 1661.2, 2093	1400,1669,2102	1393, 1673, 2104	1393, 1673, 2104
7.	Minor F#	F#	F6#, A6, C7#	1480, 1760, 2217.5	1470,1758,2245	1478, 1762, 2239	1479, 1761, 2239
8.	Minor G	G	G6, A6#, D7	1568, 1864.7, 2349.3	1561,1868,2366	1569, 1871, 2362	1566, 1873, 2361
9.	Minor G#	G#	G6#, B6, D7#	1661.2, 1979.5, 2489	1672,1981,2533	1674, 1982, 2528	1673, 1981, 2528
10.	Minor A	A	A6, C7, E7	1760, 2093, 2637	1755,2102,2667	1763, 2104, 2665	1761, 2102, 2664
11.	Minor A#	A#	A6#, C7#, F7	1864.7, 2217.5, 2793.8	1868,2242,2826	1871, 2237, 2830	1870, 2238, 2827
12.	Minor B	B	B6, D7, F#7	1979.5, 2349.3, 2960	1981,2366.2985	1982, 2361, 2991	1981, 2361, 2991

**Table 3:** Percentage error detection for Minor Chords of Piano with FFT, Morlet Wavelet and Modified Morlet Wavelet.

Sl. No.	File Name	Chord Played	Notes Present	Frequencies of notes (Theoretical) (Hz)	% Error of frequency detecting using FFT	% Error of frequency detecting using MW	% Error of frequency detecting using MMW
1.	Minor C	C	C6, D6#, G6	1046.5, 1244.5, 1568	0.13%	0.10%	0.07%
2.	Minor C#	C#	C6#, E6, G6#	1108.7, 1318.5, 1661.2	0.41%	0.29%	0.27%
3.	Minor D	D	D6, F6, A6	1174.7, 1396.9, 1760	0.40%	0.15%	0.13%
4.	Minor D#	D#	D6#, F6#, A6#	1244.5, 1480, 1864.7	0.15%	0.19%	0.17%
5.	Minor E	E	E6, G6, B6	1318.5, 1568, 1979.5	0.38%	0.19%	0.14%
6.	Minor F	F	F6, G6#, C7	1396.9, 1661.2, 2093	0.47%	0.41%	0.41%
7.	Minor F#	F#	F6#, A6, C7#	1480, 1760, 2217.5	0.63%	0.36%	0.36%
8.	Minor G	G	G6, A6#, D7	1568, 1864.7, 2349.3	0.44%	0.31%	0.36%
9.	Minor G#	G#	G6#, B6, D7#	1661.2, 1979.5, 2489	0.98%	0.97%	0.94%
10.	Minor A	A	A6, C7, E7	1760, 2093, 2637	0.62%	0.59%	0.50%
11.	Minor A#	A#	A6#, C7#, F7	1864.7, 2217.5, 2793.8	0.77%	0.76%	0.72%
12	Minor B	B	B6, D7, F#7	1979.5, 2349.3, 2960	0.55%	0.55%	0.53%

It is observed that the percentage of error detection remains minimum for the modified morlet wavelet than the FFT and MW in most of the cases.

## 4 Results and conclusions

The salient points are concluding from wavelet based multipitch estimation in polyphonic music as the work described for pitch estimation in polyphonic signals which can be easily used for monophonic signals too. For the polyphonic music, two wavelets, morlet and modified morlet wavelet are used. Algorithm uses CWT coefficient calculation at multiple scales and those scales are finally mapped to the frequencies which are having largest local peak coefficient. The algorithms were tested for the Piano chord and Standard music IOWA data base in polyphonic music signals. The obtained frequency with the algorithms has been tested and found that the wavelet can track the frequencies present in the simulated signal. For the modified MW it has been considered that the wavelet should have smaller bandwidth and Gaussian like curve in the frequency domain so that if at any scale the frequency is close to the scale, which represents the similar frequency, coefficient should have maximum value. The performance of modified MW based MPE scheme has been compared with Fast Fourier Transform and Morlet Wavelet based MPE schemes, in terms of percentage Gross Pitch Error (GPE). The results obtained with both the wavelets were very close and that was verified with the theoretical frequencies present in the chord. The computational complexity of the FFT is lower than proposed algorithm but the proposed algorithm gives better accuracy than FFT.

## Declarations

### Author contribution statement

Conceived and designed the experiments; Performed the experiments; Analyzed and interpreted the data; Contributed reagents, materials, analysis tools or data; Wrote the paper.

N. Kumar: Conceived and designed the experiments; Performed the experiments; Contributed reagents, materials, analysis tools or data; Wrote the paper.

R. Kumar: Analyzed and interpreted the data; Wrote the paper.

### Funding statement

This research did not receive any specific grant from funding agencies in the public, commercial, or not-for-profit sectors.

### Competing interest statement

The authors declare no conflict of interest.

### Additional information

No additional information is available for this paper.
